# Facts of Vertigo in Adolescents: Controversies and Challenges – A Narrative Review

**DOI:** 10.7759/cureus.28294

**Published:** 2022-08-23

**Authors:** Melissa Castillo-Bustamante, Mariana Barona Cabrera, Sara Suárez Angulo, Mariana García Campuzano, Alejandro García, Jorge Madrigal

**Affiliations:** 1 Otolaryngology, Medical School, Health Sciences School, Universidad Pontificia Bolivariana, Medellín, COL; 2 Otoneurology, Centro de Vertigo y Mareo, Medellin, COL; 3 Otoneurology, Centro de Vertigo y Mareo, Mexico City, MEX; 4 Otolaryngology, Medical School, Health Sciences School, Universidad Pontificia Bolivariana, Medellin, COL; 5 Otolaryngology, Massachusetts Eye and Ear Infirmary, Boston, USA

**Keywords:** benign paroxysmal positional vertigo, puberty, vestibular migraine, vestibular disorders, adolescents, vertigo

## Abstract

Vertigo is a common complaint in the general population affecting 5% of adults in one year. At least 29.5% of adults have referred vertigo during life. Even though the prevalence of vertigo is well known in adults the epidemiologic data in adolescents is sparse. To date, it is known that adolescent females are usually affected by vertigo and some conditions such as depression and anxiety are found in this population. However, the lack of information about the prevalence of most common types of vertigo in adolescents, predisposing factors, challenges, and controversies in clinics in the literature, present a challenge for clinicians regarding the approach and follow-up of this population. Herein, we performed a literature review including data about the prevalence, most common types of vertigo and controversial events in the approach of vertigo in adolescents over the last two decades.

## Introduction and background

Vertigo is defined as the illusion of internal or external motion and is commonly described as a sensation of spinning or swaying or having something surrounding or presenting sway [1]. This symptom occurs with considerable frequency in adolescents and may result in delayed postural control, lack of coordination, and development of paroxysmal head tilt in young patients [2,3]. Vertigo is similarly common in adolescents and in younger children, prevalence rates are estimated similarly ranging from 8% to 10% [4-6]. This symptom could be challenging to diagnose for healthcare providers, as adolescents present vertigo as a part of a complex of symptoms usually associated with viral illnesses, central nervous disorders, cardiovascular diseases, intracranial tumors, or mixed peripheral-central syndromes [7]. Also, the approach to vertigo in adolescents presents several challenges in anamnesis, physical examination, and testing [8]. Potential confounders and deviances during the clinical evaluation include the accuracy of clinical descriptions, difficulties when describing symptoms, lack of information from patients, miscommunication between parents and adolescents, and anxiety at the time of evaluation of health providers [8]. Other challenges in the evaluation of vertigo in adolescents are the increasing rates of somatoform and psychiatric disorders, which makes it even more difficult for the certain diagnosis of this symptom [2]. Acute vertigo episodes in adolescents are often managed by pediatricians and cardiologists who are the first primary care providers to evaluate this population, carrying out several cardiologic, metabolic, and radiologic testing before further management by other specialists such as otolaryngologists and neurologists [7]. This may represent a delayed opportunity for further examination such as vestibular and audiological testing and for an extensive neuro-otologic evaluation [7]. Vertigo causes a significant impact on the psychophysical and emotional health of adolescents and may require a multidisciplinary approach including diverse healthcare providers such as otolaryngologists, neurologists, psychologists, cardiologists, and ophthalmologists to unravel the etiology of vertigo and establish a proper diagnosis and treatment of this symptom [2]. Herein, we have performed a literature review of vertigo in adolescents focused on current trends, controversies, and challenges in the approach and diagnosis of this symptom in adolescents.

## Review

Methodology

The literature search was conducted in PubMed, Scopus, EBSCO, Google Scholar, and Scielo to search articles published between January 2012 and January 2022 in the English language using the following medical subject headings (MeSH) terms: “vertigo”, “adolescent”, “dizziness” and “vestibular diseases”, and Boolean operators AND/OR.

The MeSH keywords used were: Vertigo OR ((“Vertigo” [Mesh] OR “Dizziness” [Mesh]) AND Adolescent AND (“Adolescent” [Mesh]), Vestibular diseases OR ((“Vestibular diseases” [Mesh] AND Adolescent AND (“Adolescent” [Mesh]).

This review was conducted between February and May 2022. The inclusion criteria for this search included articles reporting adolescent patients, defined adolescent as the individual from ages 10 to 19 according to the definition of World Health Organization (WHO) with dizziness and/or vertigo [9]. Case reports, retrospective chart reviews, cross-sectional studies, case-control studies, cohort studies, and systematic reviews were included. Additionally, articles reporting vestibular migraine, motion sickness, benign paroxysmal positional vertigo, benign paroxysmal vertigo of childhood, and other presentations of peripheral vertigo during adolescence were included. Editorials, narrative reviews, scope reviews, letters to the editors, comments, and abstracts were excluded. Articles not focused on vertigo during adolescence and those focused on vertigo before 10 years of age and after 19 years of age were also excluded. Reports of vertigo in adolescents associated with arteriovenous malformations, inner ear malformations, tumors and central nervous system diseases were also excluded. A total of 739 articles were screened for relevance, 618 were removed due to irrelevant titles and abstracts and 64 reports were excluded due to inclusion criteria.

In total 57 indexed papers were found, of which 43 articles were duplicates, hence they were removed. Fourteen articles were included in this review. All articles selected were cross-checked by the authors. Articles were examined and classified according to the information regarding epidemiology, etiology, predisposing factors, diagnoses and potential controversial events about clinical manifestations and diagnosis.

Of the 14 studies found, 13 were retrospective and one was prospective. Of these studies three were focused on epidemiology, seven documented types of vertigo, six on clinical manifestations, and five presented some topics on controversies. The quality of evidence in the published articles was reviewed according to the 2009 Levels of Evidence of the Oxford Centre for Evidence-Based Medicine.

Results

Current Data About Epidemiology

Adolescence is a transitional stage of physical and psychological development and is generally described as the period between the dependency of childhood and the independence of adulthood [10]. Therefore, it is often assumed that adolescents show characteristics of both children and adults [10]. Traditionally, the spectrum of causes of vertigo in adolescents seems to be similar as in adults, but several studies have shown to differ from this assumption [11]. Vertigo seems to be a rising phenomenon in populations over 10 years old [11]. Over the last decade, the prevalence in the United States was described as between 0.4% to 15% [12]. At least 5-10% of adolescents were found to have at least one episode of vertigo before the age of 10 years, with a higher prevalence between 8 and 10 years, possibly due to the beginning of hormonal changes in the female population [3]. At 15 years of age, 18% of adolescents have referred at least one episode of vertigo, while 5.3% have presented more than three episodes [11,12]. Between 15 and 18 years this prevalence remains stable [11,12]. Females are usually affected by vertigo during puberty and adolescence with a peak of presentation between 10 and 19 years of age; however, the prevalence may vary according to specific disorders such as vestibular migraine, benign paroxysmal positional vertigo (BPPV), benign paroxysmal vertigo of childhood (BPVC), psychogenic vertigo, orthostatic vertigo and motion sickness.

One of the biggest surveys (800 subjects) performed in Germany, showed the prevalence of vestibular disorders in adolescents and children [11]. Benign paroxysmal vertigo (BPV) of childhood (18.7%) and vestibular migraine (17.6%) were the most frequent diagnosis, both related to migraine (40%) [11]. Other causes associated to vertigo in adolescents were trauma (14%), motion sickness (7%), central vertigo (19.1%), psychiatric disorder (12%), side effects from medications (11%), hemodynamic disorders (10.9%) and non-classified disorders (40.8%) [13-16]. However, these are not the only etiologies known, viral infections and otitis media, chronic daily headache, intracranial tumor, epilepsy, demyelinating disease, and psychogenic vertigo are also listed [7]. At least 14% of adolescents have reported at least one episode of vertigo during adolescence and 4% reported three or more episodes during the same period [11]. Adolescents experience vertigo predominantly at between 11 and 15 years of age, with a peak of presentation at 12 years of age [11].

Some types of vertigo are known to be most common in females than in men such as orthostatic dizziness (58.6%), swaying vertigo (15.0%), and unspecified dizziness (17.1%) [7,16]. No differences have been found in spinning vertigo between females and males [11]. In one cross-sectional study, at least 50% of students with either spinning vertigo or swaying vertigo were found to have orthostatic dizziness, meanwhile, those with spinning and swaying vertigo were only reported in 29.5% [11]. According to vertigo duration, adolescents seem to mostly present episodes of less than 1 minute (64.2%) [11]. Other types of duration mostly found were between 30 minutes and seven days (17.5%) [7,11]. In those vertigos with less than 1 minute of duration, spinning vertigos were commonly described while those with a duration between 30 minutes and seven days were mostly described as swaying vertigo [7,11].

The prevalence of each vestibular disorder during adolescence is highly variable between females and males [16]. To date, in adolescents, vestibular migraine is usually reported (16.2-32%), followed by benign paroxysmal positional vertigo (10-19.8%), and benign paroxysmal vertigo of childhood (8.5-24.2%) in patients ranging from 10 to 18 years old [15,16]. Even though these mentioned above are the most common types of vertigo found in adolescents, persistent postural-perceptual dizziness (PPPD) presents a rising prevalence in this population. In one retrospective review, 53 adolescents diagnosed with PPPD reported a mean age of 14.6 years [15]. In addition to PPPD diagnosis, other concomitant types of vertigo were found such as benign paroxysmal positional vertigo (64.2%), vestibular migraine (56.6%), and anxiety (28.3%) [14]. A high proportion of patients (43.4%) reported initially missing school or work due to their symptoms [7,11].

Pathophysiology and Clinical Facts During the Adolescence

During adolescence, several pathways continue their development such as the white matter in the prefrontal cortex, limbic system, and subcortical structures, all of them involved in executive, attention, and regulatory functions [17]. In this period of life, the trajectories of the limbic system and subcortical structures such as basal ganglia present a different pathway and explain the impulsivity and risk-taking behavior that adolescents usually present. Other structures such as the prefrontal cortex, increase their functioning over this time and this is clinically seen in an improved ability to suppress impulses and better emphasis on goal-driven choices [17]. Besides these facts that may be involved in some events in daily life, sports activities, school behaviors, and increased performances of risky activities and experimentation that may drive to falls, concussions, unsteadiness, and altered proprioception, the vestibular system in this lifetime still being under development until age 16 [18]. In adolescents, the late functional development of the vestibular system alters somatosensory and visual control of balance leading to common disorders in this lifetime such as motion sickness and increased rates of visual vertigo [18,19]. As pathophysiologic events happen, there are other external factors that are also involved in vertigo during adolescence [18,20]. One is the asthenic habitus of adolescents, increased stress and sleep deprivation, higher rates of migraines associated with hormonal changes, and muscular pain in the neck and shoulder region [20]. From this perspective, ongoing changes in the vestibular system, differential functioning pathways involved in adolescents’ behavior, altered somatosensorial control, and external factors potentiate vertigo in adolescents [20].

Regarding the clinical presentation of vertigo, the main concomitant complaints of adolescents during clinic visits reported in three retrospective studies were headache (51%), nausea (8%), and vomiting (2%) [21]. Other complaints during the clinical visit were intolerance to head movements and positional changes, mainly when turning the head, sitting and laying down, and practicing sports [15]. Physical activity and concussion are usually associated with vertigo in adolescents with increasing rates over the last years. In one retrospective review, at least 29.4% of teenagers were diagnosed with BPPV. These presented a mean age of 14.7 ± 3.4 years (range 7-20 years), females and males did not show any difference in their presentation, and most of them reported concussion after physical activity (34%) [21,22].

Other less frequent symptoms related to vertigo were unsteadiness while standing for long periods and walking [9]. Other events associated with vertigo included detailed triggers such as stress, muscle pain in the neck and shoulders, sleep deprivation, migraine, and alimentary disorders [23]. In the evaluation of HINTS protocol (Head Impulse, Nystagmus and Test of Skew), one prospective study revealed the presence of a positive head impulse (14%) and spontaneous nystagmus (27%) as the most common findings [23]. Other findings reported were post-headshaking nystagmus and positional nystagmus as a complementary result in the videonystagmography [22]. During the performance of Romberg and Unterberger Test, no pathological findings were reported in three studies [22]. Using HINTS-Plus in adolescents, only one study reported after vertigo episodes, high-frequency hearing loss in six patients (16%), and low-frequency hearing loss in three patients (18%) [22]. Only one patient in this study described unilateral profound hearing loss associated with the debut of sudden hearing loss and vertigo [22]. One retrospective study was also shown in teenagers with vertigo abnormal testing in a rotary chair, caloric testing, and video head impulse test as well as the subjective visual vertical mainly in types of sudden vertigo such as vestibular neuritis and vestibular migraine [24].

Common types of vertigo and new challenges in the clinic

Vestibular Migraine

Vestibular migraine is one of the most common types of vertigo, frequently seen in females since puberty and described in the literature as the most relevant type of vertigo during adolescence in females and males [25-28]. Endogen and exogen estrogens have been studied to affect the function of the vestibular system [29]. There are previous studies reporting the presence of estrogen receptors at the stria vascularis and spiral ligament [29]. It is possible that estrogen fluctuations could control inner ear homeostasis, regulation of the endolymphatic fluid, and changes in electrolytic ionic and anionic function [29]. These hormonal fluctuations in women occur mainly during the menstrual cycle, pregnancy, and menopause, in which changes in the homeostasis of labyrinthine fluids can be generated, with a direct influence on enzymatic processes and neurotransmitter action [30]. The variation in the behavior of the labyrinthine fluids, as well as the interference in the sensitivity of the enzymatic receptors, influences the basal metabolism of the inner ear, thus justifying otological symptoms in women [29]. Vestibular migraine is nowadays a well-known entity that can occur at any age; in adolescents, this type of vertigo is now recognized by the Barany Society, targeted in females and males coming up from childhood to 18 years of age [29]. Regarding migraine headache and vertigo characteristics, there is only one reported variation in presentation, where adolescents more often present with bilateral migraine headache than adults [28]. This disease may present some difficulties in its diagnosis because of overlapping symptoms and other comorbidities such as psychiatric disorders, exacerbation of PPPD, or generalized anxiety disorder, that also may cause autonomic and vestibular symptoms [30]. One of the most important challenges in adolescence besides the presence of vestibular migraine is the concomitant presentation of motion sickness which can also be misdiagnosed and superposed to the presentation of vestibular migraine [30]. This usually happens due to disturbances in the visual system, cues for spatial orientation, and a disability to integrate multisensory input to resolve sensory conflicting situations that may provoke imbalance and vertigo [30]. Motion sickness represents a challenge for the diagnosis of vestibular migraine. Usually, motion sickness presents before puberty and in other cases, increases after menarche or appears concomitantly to the onset of vestibular migraine [29,30]. During adolescence, there is one additional challenge when diagnosing vestibular migraine, which is the presence of episodic ataxia grade II, an autosomal dominant disorder usually presented under age 20, which includes episodic attacks that last from hours to days and could be precipitated by stress or exercise, headache, vertigo, hereditary and periodic vestibulocerebellar ataxias, nausea and vomiting and includes a mutation in the subunit P/Q type calcium channels located in chromosome 19p [31,32]. The main challenge with these symptoms is the similarity with the episodes of vestibular migraine, which can overlap some of them and may mimic this type of ataxia [33,34]. Magnetic resonance in challenging cases may be useful, as in this imaging examination the atrophy of cerebellar vermis is a critical finding [34]. Further examinations are needed before establishing the diagnosis of vestibular migraine, starting with an extensive anamnesis and clinical history recording.

Benign Paroxysmal Positional Vertigo

Benign paroxysmal positional vertigo is often found in adolescents [35]. Some specific events during sports and daily activities such as head hanging positions and concussion during competitive sports may trigger this disorder. Other situations associated with benign paroxysmal positional vertigo are inner ear anomalies and otologic and cranial surgery due to the use of high-speed drills [35]. Besides the maneuvers, some specific testing such as a video-head impulse test and a brief examination of vestibule-ocular reflex using Frenzel goggles should be performed to confirm the presence of benign paroxysmal positional vertigo during the approach of the adolescent patient [35]. Benign paroxysmal torticollis of infancy and benign paroxysmal vertigo of childhood may be confused with benign paroxysmal positional vertigo for healthcare professionals, leading to some directional mistakes in therapeutic and follow-up [35]. This represents a challenge, as these disorders usually progress to vestibular migraine (Table 1). It is necessary to truly understand that these entities are not the same ones, and the correct diagnosis is critical to understand its progression from childhood to adolescence [35].

**Table 1:** Vestibular disorders presenting vertigo in adolescents according to age and gender

Vestibular disorders presenting vertigo in adolescents	Studies	n	Mean age (SD)	Vertigo (%)	Women, n (%)	Men, n (%)
Vestibular Migraine	Gruber et al. [23]	37	14 ± 3.3	32	-	-
Benign paroxysmal positional vertigo	Balatsouras et al. [8]	54	8.9 ± 4.2	16.7	5 (55.5)	4 (44.4)
	Erbek et al. [21]	50	6.2 ± 4	12	3 (50)	3 (50)
	O'Reilly et al. [22]	132	9.7 ± 5	2.2	-	-
	Brodsky et al. [35]	110	13.4 ± 3.4	19.8	-	-
Psychogenic vertigo	Erbek et al. [21]	50	6.2 ± 4	12	3 (50)	3 (50)
	O'Reilly et al. [22]	132	9.7 ± 5	24.2	-	-

Vestibular Paroxysmia

Vestibular paroxysmia is one of the newest diagnoses in adolescents and is usually described as a disorder in the pediatric and adolescent population. Its etiology is attributed to a neuro-vascular cross-compression syndrome and narrowed internal auditory canal (IAC), which appears to be involved in the development of vertigo and balance impairment leading to the compression of the VII, VIII cranial nerves and the labyrinthine artery. The prevalence of this type of vertigo is unregistered but its presentation is usually seen in late adolescence [36]. Clinical manifestations are described as frequent vertiginous spells that last seconds to minutes, occur at rest and with certain head positions, and respond to treatment with low-dose sodium channel-blocking [36-41]. The challenge with this type of vertigo is the similarity with the benign paroxysmal positional vertigo. Herein, it is necessary to perform maneuvers with Frenzel goggles as well in difficult cases of vestibular testing such as videonystagmography and video-head impulse test [36]. When magnetic resonance is useful while performing the diagnosis, this is done concomitantly with a supplementary HRCT of temporal bones with an analysis of the shape, diameter, and opening width of the IAC in both axial and coronal planes [36-41].

Persistent Postural-Perceptual Dizziness (PPPD)

PPPD is one of the most common and treatable causes of chronic dizziness in the pediatric population [42]. This disease is associated with an impairment in quality of life, including a reduction in school attendance and sports practice. This disorder has been increasing over the time, and nowadays there is a prevalence in the pediatric and adolescent population of 7.3% [42]. Symptoms associated with this disorder are on-vertiginous dizziness and a sense of imbalance exacerbated by position changes, standing, turning, and visual flow. This disorder may last for many months and even years before receiving diagnosis and treatment [42]. Another important fact of this disorder is the concomitant presence of anxiety and depression in this population, with higher rates of neuroticism and low extroversion as well as psychological chronic stress led by autonomic amygdala response to feared physical sensations such as pain or dizziness [42].

Somatoform Vertigo or Psychogenic Vertigo

Somatisation is a prevalent condition in adolescents and may be an expression of other underlying psychiatric problems [43]. This is defined as the expression of multiple somatic symptoms that are not explainable medically but are associated with psychological stress and medical help-seeking. This condition causes an impairment in quality of life and usually presents another underlying psychiatric problem such as anxiety and depression [43]. The current prevalence is estimated in 1.1% of adolescents, however, still being difficult to study because of the lack of appropriate diagnostic criteria [43]. Besides this challenge in diagnosis somatisation with normal daily functioning in children and adolescents seems to be a more common phenomenon and adequate interdisciplinary management seems difficult due to the lack of fully trained professionals in this setting [43]. Somatisation should be considered when dealing with adolescents with vertigo; meticulous evaluation using clinical evaluation, vestibular testing, and even imaging tools are recommended and as soon as the possibility of somatisation comes to clinicians, psychiatric consultation is recommended [43].

Other causes of vertigo to consider

Other common causes of vertigo found in younger patients than 16 years old are viral labyrinthitis, migraine, psychogenic and viral infections, or otitis media [7]. Some more common causes are chronic daily headache, trauma, and postural orthostatic tachycardia syndrome, and less common causes are intracranial tumors, epilepsy, Meniere’s disease, vestibular neuritis, and demyelinating disease [7]. These disorders have been previously exposed in one of the most complete reports about vertigo and dizziness in adolescents and are not the aim of this study [7].

Considerations on vestibular testing

Video head impulse test and vestibular-evoked myogenic potentials test are used for quantifying vestibulo-ocular reflex and utricular and saccular otolith functions and are purposed as diagnostic tools for the evaluation of adolescent patients with vertigo [7]. Its application is still under study and remains controversial. Some preliminary studies have shown that it is useful to detect even mild semicircular canal function deficits, however, there are few professionals fully trained in this evaluation method [7]. Instead, caloric testing on the videonystagmography and rotation chairs are described as useful diagnostic testing on adolescents, but their use reports unpleasant vertigo and is not well tolerated [7]. Vestibular-evoked myogenic potential testing is more often considered in adolescent patients with special conditions such as those with hearing impairments, and faulty cochlear implants and it is used to detect impaired motor development as well as to detect other specific deficits such as congenital torticollis and to determine susceptibility to motion sickness [7].

Magnetic resonance is usually requested under some specific conditions such as head trauma, new onset of vertigo with or without headache, propensity for risk-taking behavior, and concussion in order to assess the presence of skull fracture [7]. Other indications are to assess for cranial abnormalities, suspicious inner ear abnormalities, and diffuse axonal injury seen after high-speed collisions and labyrinthine concussions or central lesion [7].

Vertigo in adolescents is common but there are some challenges in its approach. First, the description of symptoms of this population, which may be sometimes not concise and can omit several details [20]. Another challenge is the lack of empathy between parents, clinicians and patients, miscommunication, and distractibility when characterizing events and episodes.

Limitations of this narrative review are the lack of articles related to this topic, no cohorts and randomized controlled studies available and the small number of studies included. Further studies focused on therapeutics, vestibular rehabilitation, and other alternative treatments are needed for future and more extensive reviews.

## Conclusions

Vertigo in adolescents is a common underreported complaint in this population. Females are most frequently affected, and vestibular migraine is the most common type of vertigo found. Estrogen fluctuations may be associated with its pathogenesis. Improving the understanding of vertigo in this population will allow healthcare professionals to give a critical and focused diagnosis, follow-up, and rehabilitation.
